# The low-profile loop titanium plate system for acromioclavicular joint dislocation: A retrospective cohort study on learning curve and clinical efficacy

**DOI:** 10.1097/MD.0000000000044885

**Published:** 2025-10-10

**Authors:** Liang Zhang, Jin Zhang, Longhui Shao, Jing Li, Chunling Song, Juan Bai, Lei Wang

**Affiliations:** a Department of Orthopedics, The Fifth People’s Hospital of Ningxia, Ningxia, China; b Department of Nursing, Integrated Traditional Chinese and Western Medicine Hospital of Ningxia Fifth People’s Hospital, Ningxia, China.

**Keywords:** acromioclavicular joint dislocation, clinical efficacy, cusum curve, hook plate, learning curve, low-profile loop titanium plate system

## Abstract

This study aimed to evaluate the clinical efficacy of LP-LTPS for treating Rockwood type IIIB ACJ dislocation, while analyzing the learning curve and CUSUM curve of the new technique. A retrospective analysis was conducted on 179 patients with Rockwood type IIIB ACJ dislocation (82 in the LP-LTPS group and 97 in the hook-plate control group) between April 2020 and May 2022. Learning curve and CUSUM curve analyses were performed using operation time data from the LP-LTPS group, with the first 5 cases excluded as the learning phase. Clinical outcomes included Constant-Murley Score and visual analogue scale, operation time, incision length, blood loss, complications, and radiographic parameters. Statistical comparisons used independent t-tests for continuous variables and chi-square tests for categorical variables (*P* <.05). The learning curve showed surgical proficiency was achieved after 5 cases, with stable operation time (38.88 ± 5.42 minutes). The LP-LTPS group demonstrated significantly shorter incision length, reduced blood loss, and lower complication rate versus the control group (all *P* <.05). Postoperative Constant-Murley Score was higher in the LP-LTPS group at all time points (*P* <.05). For visual analogue scale, the LP-LTPS group had significantly lower pain scores at 2 days, 1 month, and 3 months postoperatively (*P* <.001), with no significant differences at 6 and18 months (*P* >.05). Preoperative AC/CC distances were comparable between groups (*P* >.05). At 12 months postoperatively, both groups showed significant reductions in AC/CC distances versus baseline (*P* <.001), with no significant between-group differences (*P* >.05). LP-LTPS offers superior clinical outcomes with a short learning curve, fewer complications, better functional recovery, and early pain control compared to control group for Rockwood type IIIB ACJ dislocation, supporting its use as an effective alternative.

## 1. Introduction

Acromioclavicular joint dislocation(ACJ) is a prevalent shoulder injury, typically resulting from direct trauma to the shoulder.^[1]^ This injury leads to joint instability, causing substantial pain and functional impairment, which significantly affects patients’ daily activities and quality of life.^[2–4]^ Surgical intervention is often indicated for patients with Rockwood type III or higher ACJ, as these intermediate to severe classifications represent the most common indications for ligament reconstructive techniques.^[4,5]^

The hook plate has been a traditional treatment modality. It functions by hooking beneath the acromion and fixing to the clavicle, providing a certain level of stability to the acromioclavicular joint.^[6–9]^ However, follow-up studies have revealed a series of complications associated with this method, such as subacromial impingement and hook-plate fracture.^[10,11]^ In contrast, the low-profile loop titanium plate system (LP-LTPS)represents a simplified, self-tensioning anatomical reduction process, designed to reconstruct the coracoclavicular ligament and offer nonrigid fixation.^[5,8,10,12,13]^ This device represents the principle of low-profile fixation with a thinner design, smoother edge transitions and tensile strength load. These features are specifically engineered to minimize soft tissue irritation and fixation loosening.^[14,15]^ By effectively conforming to the anatomical structure of the clavicle, it significantly reduces friction with muscle tissues. This not only potentially decreases the occurrence of complications related to fixation but also promotes better functional recovery for patients.

In this study, we first focused on the learning curve and Cumulative Sum (CUSUM) curve before evaluating the clinical efficacy of LP-LTPS in patients with Rockwood type IIIB ACJ. The learning curve and CUSUM curve reflect the process by which surgeons become more proficient in performing a new surgical technique over time.^[16–18]^ Moreover, they reflect surgical difficulty – understudied yet critical, lower difficulty fosters technique spread and better clinical outcomes. Understanding the learning curve and CUSUM curve for LP-LTPS is crucial prior to evaluating its clinical efficacy. These analyses allow for the exclusion of data from the initial learning phase, ensuring that comparisons of postoperative outcomes between LP-LTPS and established techniques (the hook plate) are not confounded by the surgeon’s early inexperience with the new system. This approach provides a more accurate and reliable assessment of the true clinical performance of LP-LTPS. Therefore, we first analyzed the learning curve and CUSUM curve for LP-LTPS before evaluating other outcome measures. This approach ensured that the data used for comparing the 2 treatment methods (LP-LTPS vs hook plate) were not confounded by the inexperience of the surgical team during the initial stages of adopting the new technique. The results of these curve analyses were used to exclude data from the early, potentially less-proficient surgical cases, providing a more accurate and reliable comparison between the 2 techniques in terms of postoperative pain, functional recovery, and patient satisfaction. This ultimately provided more evidence-based medical information for clinical decision-making in the treatment of ACJ.

## 2. Patients and methods

### 2.1. Study subjects

In compliance with the Declaration of Helsinki and approved by the Ethics Committee of our hospital (approval number: NXWY2025GK0420), a cohort study was conducted on patients with ACJ admitted to our hospital between April 2021 and May 2023.

Inclusion criteria were as follows: definite diagnosis of Rockwood classification of IIIB ACJ based on clinical symptoms, physical examination, and confirmatory imaging findings; age between 18 and 60; injury occurred within 10 days before surgery; and patients provided informed consent.

Exclusion criteria: concomitant fractures or dislocations in other parts of the shoulder; previous shoulder surgery; severe osteoporosis; and significant comorbidities that could affect outcomes, specifically: uncontrolled diabetes mellitus, active local or systemic infection, rheumatoid arthritis with shoulder involvement, neurological disorders affecting shoulder function, and severe cardiopulmonary disease contraindicating surgery.

All methods were performed in accordance with the Declaration of Helsinki. Informed consent was obtained from all subjects and/or their legal guardians.

Sample size: Using G*Power software to assume a medium effect size (a moderate difference in Constant-Murley Score(CMS), Cohen d = 0.5), with α = 0.05 (two-tailed) and power = 0.80, each group required at least 64 patients.

Demographic and baseline characteristics: Collected variables included age (mean ± SD, range), gender (male/female counts), affected side (left/right distribution with counts and percentages).

Finally, a total of 179 patients were included, with 82 patients in the LP-LTPS group(study group)and 97 patients in the hook-plate group (control group). (*P* >.05, Table 1). Baseline characteristics (age, gender, side, injury type) were compared using independent t-tests for continuous variables and chi-square tests for categorical variables.

**Table 1:** Comparison of baseline characteristics and surgical parameters between study group and control group.

	Study group (n = 77)	Control group (n = 97)	*P*-value
Age (year)	43.3 ± 9.91	42.43 ± 11.33	.689
Gender (M/F)	30/47	42/57	.643
Side (L/R)	38/39	50/47	.774
Injury type			
Sports-related	24	36	.535
Traffic accident	21	25	
Fall-induced	32	36	
Incision length (cm)	5.46 ± 1.100	6.38 ± 1.064	<.001
Operation time (min)	38.88 ± 5.42	54.00 ± 11.73	<.001
Blood loss (mL)	15.00 ± 5.06	44.38 ± 4.20	<.001
Complication	2	11	.03
Subacromial erosion	0	4	–
Reduction loss	2	4	–
Peri-implant fractures	0	2	–
Vascular/nerve injury	0	1	–
Incision infection	0	0	–
Other	0	0	–

### 2.2. Surgical procedures

**Figure 1.**
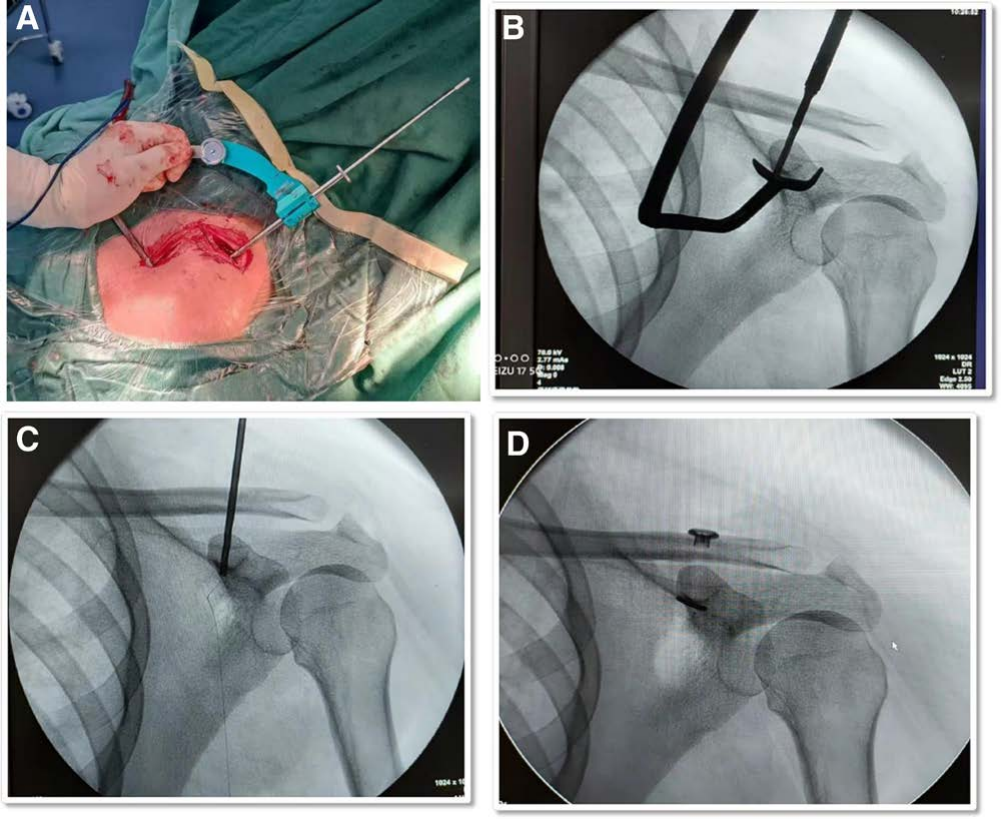
The picture and X-ray of a 49-year-old man with Rockwood type IIIB ACJ injury treated by the low-profile loop titanium plate system. Panel (A) shows the surgical incision and localization of the coracoid positioner; (B) shows X-ray image of Coracoid positioner aligned with the base of the coracoid process under fluoroscopic guidance; (C) shows X-ray image of the establishment of bone tunnels; (D) shows X-ray image of anatomical reduction with the low-profile loop titanium plate system. ACJ = acromioclavicular joint.

For patients in LP-LTPS group (Study Group), the procedure was performed under general anesthesia with the patient in the beach chair position. Two incisions were made: a 15 mm transverse incision 30 mm proximal to the acromioclavicular joint and a 20 mm longitudinal incision over the coracoid process. The coracoid process locator (an angled instrument with a hook-shaped distal end and a handle) was used as follows (Fig 1A):

1. Locator alignment: The locator’s handle was locked at a 90° angle to the hook-shaped tip to ensure rigid stability. The locator was then inserted through the coracoid incision and positioned at a 40° angle from medial to lateral, with the hook contoured to the lateral base of the coracoid process.

2. Anatomical validation: Surgeons confirmed proper placement by palpating the locator’s fit against the coracoid base to ensure no gaps or misalignment. A fluoroscopic image was obtained to verify the locator’s position relative to the clavicle and coracoid (Fig. 1B).

3. Bone tunnel creation: With the ACJ reduced, a guidewire was drilled through the locator’s central channel to create a 4.5 mm diameter bone tunnel from the clavicle to the coracoid base. Surgeons assessed the drill’s passage through **four distinct cortical layers to confirm tunnel accuracy.

Subsequent steps included inserting the low-profile loop butterfly-shaped titanium plate through the tunnel, tensioning the polyethylene loop with alternating traction on the dual-color sutures, and securing the plate with cortical compression. The ACJ reduction was confirmed by direct visualization and fluoroscopy before closing incisions (Fig. 1C and D).

Patients in the Hook-Plate Group (Control Group) received conventional clavicular hook-plate surgery treatment.

### 2.4. Postoperative management

All patients initiated passive pendulum exercises of the affected shoulder on postoperative day 3 under physical therapist supervision, with abduction/forward flexion limited to ≤90°, external rotation ≤30°, and extension restricted. Progressive active range of motion exercises were introduced at week 2 based on clinical assessment.

Postoperative follow-up was conducted via in-person clinic visits at predefined time points, including before surgery, 2 days, 1 month, 3 months, 6 months, 12 months, and 18 months after surgery. At each visit, standardized assessments were performed: shoulder function was evaluated using the CMS; pain levels were recorded using the visual analogue scale (VAS, 0–10, with 0 indicating no pain and 10 indicating worst pain); and standardized anteroposterior and Zanca view radiographs were obtained for radiographic assessment, including measurement of acromioclavicular (AC) distance and coracoclavicular (CC) distance (with preoperative and 12-month postoperative measurements as key time points). All clinical and radiographic assessments were conducted by 2 independent orthopedic surgeons blinded to the surgical group assignment. For the hook-plate group, internal fixation was removed at 12 months postoperatively.

### 2.5. Observation indicators

#### 2.5.1. Learning curve and CUSUM curve

Analysis: Data Arrangement: In LP-LTPS group, 82 patients were sorted in the sequence of their surgeries. Only the number of surgical cases and the corresponding operation time for each case were considered in this analysis.

#### 2.5.2. Calculation of CUSUM value

The following formula was applied for calculations.


$$\begin{array}{l} CUSUM=\overset{n}{\underset{i=1}{\sum }}\left(T_{i}-\bar{T}\right) \end{array}$$

Here, T_i_ represents the operation time of the i - th single operation, ‾T is the average operation time calculated by dividing the total operation time of all cases by the number of cases n, and n represents the serial number of patients in the surgical sequence. Learning Curve Plotting: A scatter plot was created with the number of surgical cases on the x - axis and the CUSUM value on the y - axis. Then, using R package version 4.3 a curve was fitted to the scatter data points. If the *P* value was <0.05, the curve-fitting was considered successful.

Learning Phase Classification: The learning curve was categorized into a learning phase and a proficient phase. The apex of the CUSUM curve served as the dividing point. The x - axis value at this apex indicated the minimum cumulative number of cases required for surgeons to surpass the learning phase and enter the proficient phase when using LP-LTPS.

#### 2.5.3. Shoulder joint scores

CMS: This score was used to assess shoulder function before and after surgery. It includes evaluations of pain(15 points), range of motion (40 points), strength (25 points), and daily activities(20 points), with higher scores indicating better function.^[19]^

VAS: Pain score (0–10, 0 = no pain, 10 = worst pain) recorded at each follow-up.

#### 2.5.4. Operative and postoperative index

Operation Time: The duration of the surgical procedure was recorded.

Length of hospital stay: The number of days the patient remained hospitalized after surgery was noted.

Complications: Any postoperative complications, including infection, implant failure, vascular or nerve injury, rotator cuff injury and recurrence of dislocation, were recorded.

Radiological parameters: Using postoperative X-rays (anteroposterior and axillary views) at each follow-up time point: AC distance and CC distance

### 2.6. Statistical methods

Statistical analyses were performed using 3 software programs with distinct functionalities to ensure comprehensive data processing, analysis, and visualization:

R software (version4.3) was exclusively used for learning curve modeling and Cumulative Sum (CUSUM) curve-fitting. This included linear regression with natural-logarithm transformation of patient sequence numbers to model operative time trends and CUSUM value calculation for phase classification (learning vs proficient phase). Curve-fitting success was determined by a significance level of (*P* < .05), and the best-fit model was selected using the coefficient of determination (R^2^).

SPSS software (version26.0, Chicago) was used for all primary statistical comparisons: Independent samples t-tests were applied to compare continuous baseline and surgical parameters between groups (e.g., age, operation time, incision length, blood loss). Chi-square tests were used to analyze categorical variables, including gender distribution, injury type (sports-related, traffic accident, fall-induced), and complication rates. Repeated-measures analysis of variance (ANOVA) was utilized to compare longitudinal outcomes across follow-up time points, including CMSs, VAS pain scores, and radiographic parameters.

- GraphPad Prism (version 9.0) was used for data visualization, including generating histograms (Fig. 3) to compare key outcomes between groups.

**Figure 2.**
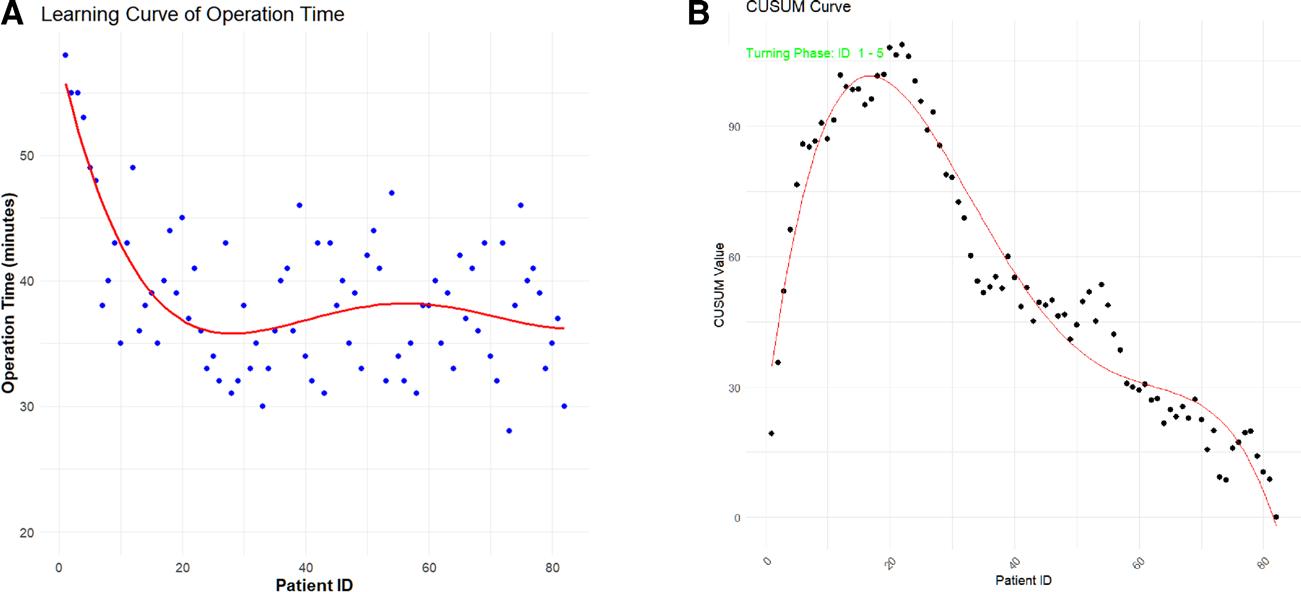
Learning curve and CUSUM curve of the low-profile loop titanium plate system group. CUSUM = cumulative sum.

**Figure 3.**
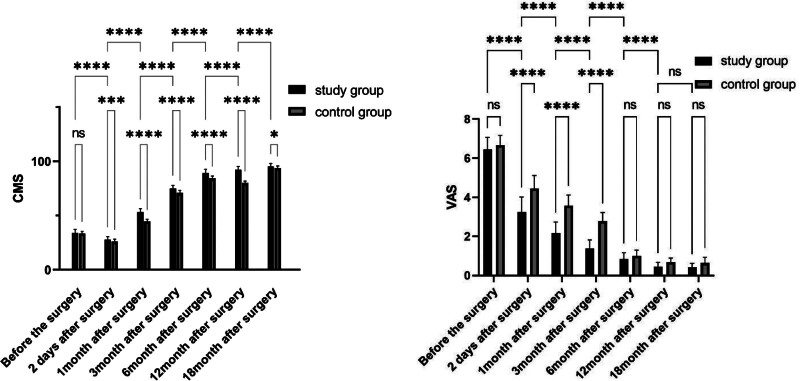
The comparison of CMS and VAS in study group and control group at different stages, ns represents *P* > .05,* represents *P* < .05,** represents *P* < .01,*** represents *P* < .001,**** represents *P* < .0001. CMS = Constant-Murley Score, VAS = visual analogue scale.

A 2-tailed (*P* <.05) was considered statistically significant for all analyses.

## 3. Results

### 3.1. Learning curve and CUSUM curve analysis

For the learning curve of operation time (Fig. 2A), We modeled this relationship using a linear regression model with a natural-logarithm transformation of the patient sequence number(R ^2^ = 0.464, *P* < .001).

the operation time initially presented a relatively high value around 55 minutes. However, with the increase in the number of patients, a clear decreasing trend was observed. The operation time greatly decreased and eventually reached a stable level after around 5 cases, during which the operative time stabilized at around 38 minutes in average.

Regarding the CUSUM learning curve (Fig. 2B), the CUSUM values could be divided into 2 phases. In Phase A (case 1–5), the CUSUM values fluctuated, reflecting the learning and adjustment process of the surgical team., the CUSUM values in entered Phase B at around 6 cases, where they became relatively stable. This transition indicates that the surgical technique had stabilized in this stage, and the surgical team had achieved a proficient level in performing LP-LTPS surgery (*R*^2^ = 0.944, *P* < .001).

### 3.2. Patient characteristics and surgical parameters

A total of 179 patients were enrolled in the study, with 82 patients in the study group (LP-LTPS treatment, 5 cases were excluded in this step given that surgeons were not familiar with this operation) and 97 patients in the control group (hook-plate treatment). Baseline characteristics (age, gender, injury type, comorbidities) were comparable between groups, ensuring baseline comparability and reducing selection bias.

The 2 groups were comparable in terms of age, gender distribution, side of injury, and Rockwood classification of ACJ (*P* >.05) for all. However, significant differences were noted in surgical - related parameters. The incision length in the study group (5.46 ± 1.10cm) was significantly shorter than that in the control group (6.38 ± 1.064cm, *P* <.001). The operation time was also shorter in the study group (38.88 ± 5.42 minutes vs 54.00 ± 11.73 minutes) compared to the control group (min, *P* <.001). Additionally, the blood loss in the study group (ml) was significantly less than that in the control group (15.00 ± 5.06 ml vs 44.38 ± 4.20 ml, *P* <.001). The incidence of complications was lower in the study group (n = 2, loss of reduction) compared to the control group (n = 11, 4 cases for subacromial erosion, 4 cases for loss of reduction, 2 cases for peri-implant fractures, 1 case for vessel injury), *P* value was 0.03 (Table 1).

### 3.3. Constant-Murley score and VAS

The CMS was utilized to assess shoulder function before and after surgery. Before surgery, there was no significant difference in CMS between the 2 groups (34.02 ± 3.19) in the study group(n = 77) vs (33.51 ± 1.91) in the control group, (*P* = .986).

After surgery, both groups demonstrated an improvement in CMS over time. Nevertheless, the study group had a significantly higher CMS than the control group at multiple time points. At 2 days after surgery, the CMS in the study group was 27.79 ± 2.765, while in the control group it was 26.67 ± 2.050, with a statistically significant difference (*P* = .007). Similarly, at 1,3, 6,12 and 18months after surgery, the study group had significantly higher CMS values (53.15 ± 3.151, 74.93 ± 4.23, 88.55 ± 4.565, 91.94 ± 3.411,95.20 ± 2.70, respectively) compared to the control group (44.62 ± 1.823 *P* < .0001, 70.27 ± 4.202 *P* < .0001, 83.54 ± 4.596 *P* < .0001, 79.89 ± 2.48793 *P* < .0001,93.76 ± 1.91 *P* = .013, respectively) for all comparisons. Within the study group, all pairwise comparisons between consecutive follow-up periods (e.g., 1 vs 3 months, 3 vs 6 months) at before the operation, 2days,1, 3, 6, 12, and 18 months postoperatively showed *P* < .0001 (Fig. 3).

VAS: Pain levels via VAS showed no significant preoperative difference: study group (6.64 ± 0.62) vs control group (6.67 ± 0.51, *P* = .812). Both groups had gradual VAS reduction postoperatively, but the study group had significantly lower scores at early time points: 2 days (3.24 ± 0.76 vs 4.40 ± 0.68, *P* < .001); 1 month (2.13 ± 0.58 vs 3.57 ± 0.55, *P* < .001); 3 months (1.39 ± 0.43 vs 2.77 ± 0.45, *P* < .001). No significant differences at 6 months (0.83 ± 0.32 vs 1.00 ± 0.30, *P* = .076), 12 months (0.45 ± 0.21 vs 0.68 ± 0.21, *P* = .052), or 18 months (0.43 ± 0.19 vs 0.64 ± 0.28, *P* = .061) (Fig. 3).

### 3.4. Radiological parameters

Preoperative AC distance and CC distance measurements confirmed comparable severity of dislocation between groups (AC distance: 9.80 ± 3.71mm vs 10.61 ± 4.30mm, *P* = .189; CC distance: 18.20 ± 5.89mm vs 19.50 ± 6.30mm, *P* = .334). Postoperative radiographic evaluation at 12 months demonstrated significant reduction in both parameters compared to preoperative values in both groups (AC distance: 3.13 ± 1.80mm vs 3.40 ± 2.14mm; CC distance: 12.25 ± 2.86mm vs 12.84 ± 3.90mm; *P* < .001 for within-group comparisons), with no statistically significant differences between groups at the 12-month follow-up. (AC distance *P* = .215, CC distance *P* = .187) (Table 2)

**Table 2:** Preoperative and 12-month postoperative radiographic parameters (AC distance, CC distance) between two groups.

	Distance (mm)	Study group (77)	Control group(97)	*P*-value
Preoperation	AC distance	9.80 + 3.71	10.61 + 4.30	.189
	CC distance	18.20 + 5.89	19.50 + 6.3	.334
12 mo after operation	AC distance	3.13 + 1.80	3.40 + 2.14	.215
	CC distance	12.25 + 2.86	12.84 + 3.90	.187

## 4. Discussion

The learning curve analysis shows that surgeons need to complete 5 cases to master LP-LTPS proficiently, and the operation time stabilizes at about 38 minutes in average. The reduction in operation time from about 55 minutes to 38 minutes reflects an improved familiarity with the bone tunnel location and a simplification of the ligament reconstruction steps. At 5th case, the CUSUM curve transitions from learning phase to stable phase, further confirming the technical consistency over time, which is crucial for clinical application.

Results indicated that surgeons attained proficiency in using LP-LTPS after 5 cases, with operative time stabilizing at 38.88 ± 5.42 minutes. This efficient learning process can be attributed to the system’s ergonomic design, including a preassembled suture-loop structure and a coracoid positioner that enables anatomical positioning. Biomechanical tests show the system’s superior performance: it exhibits 1.845mm displacement under cyclic loading, a ultimate load of 1318N, and withstands 1 million cycles in fatigue testing without failure. These results validate its mechanical stability and durability, consistent with the low complication rate observed in this study.

Also, the low-profile design (10 mm flange, 5 mm base) and dual-color suture configuration minimize soft tissue irritation and allow precise tension adjustment. The preloaded sutures and titanium plate facilitate anatomical reconstruction of the coracoclavicular ligament, with biomechanical properties mimicking the native ligament’s tensile strength (651.16N), This design reduces the risk of suture tangling and bone tunnel failure, which may explain the lower rate of implant failure,.^[20,21]^

The study group achieved significantly higher CMS at all postoperative time points, which aligns with the findings of another Chinese study that reported comparable improvements in shoulder function using a similar low-profile fixation system.^[22]^ This early and sustained functional superiority may be partially explained by reduced postoperative pain – evidenced by significantly lower VAS scores in the first 3 months – likely due to less soft tissue dissection, the absence of subacromial hardware, and more stable initial fixation.

Crucially, the early VAS advantage translated to tangible clinical benefits: This superiority may stem from the system’s nonrigid fixation mechanism, which allows 1 to 2 mm of micro-movement, simulating physiological joint mechanics and promoting ligament healing. The CMS of the study group exceeded that of the control group postoperatively, especially in the early stage of recovery. Preoperative AC and CC distances were comparable between groups, confirming balanced baseline dislocation severity. Postoperatively, both groups achieved significant reductions in AC/CC distances at 12 months (*P* < .001 within groups), validating that both fixation techniques effectively restore anatomical alignment. This suggests that the LP-LTPS system provides a stable anatomical restoration of the ACJ complex in the mid-term. The nonrigid fixation allowing for physiological micromotion likely promotes ligamentous healing in a more anatomical position

The complication rate of the study group was significantly lower than that of the control group. This difference may be due to the low-profile design reducing subacromial irritation and the precise positioning. only 2 cases of reduction loss and no vessel injuries occurred in the study group, while 11 patient in the control group experienced subacromial erosion, reduction loss, peri-implant fractures, as well as 1 vessel injury, which is consistent with the reported hook-plate fatigue rate.^[23]^ Shorter incision length and reduced blood loss further emphasize its minimally invasive advantages. The low-profile plate’s design enables broad application in orthopedics for various fractures, leveraging its proven biomechanical stability and minimizing soft tissue irritation through a thinner profile and smoother edge transitions.^[24–26]^ The fixation design of the low-profile plate shares similarities with TightRope and Endobutton, Like these systems, its design prioritizes dynamic fixation rather than rigid immobilization, while uniquely incorporating a low-profile architecture to minimize subacromial irritation. Other studies have shown the advantages of this nonrigid fixation design compared with hook-plate treatment.^[13,23,27–30]^ Recently, arthroscopic acromioclavicular joint reconstruction techniques^[27,29,31,32]^ also have gained popularity due to their minimally invasive nature, the ability to address concomitant injuries, and the elimination of the need for secondary implant removal. However, these techniques either require experienced surgeons to perform the surgeries or involve relatively high treatment costs.

LP-LTPS has a relatively low learning curve for surgeons, which means that surgeons can master the relevant surgical techniques more easily. Moreover, it does not require a second - stage surgery for implant removal, reducing the burden on patients. In addition, it can better adapt to the dynamic changes of the joint in daily activities.

### 4.1. Limitations and future directions

Despite these findings, the study has several limitations. It is a single-center, retrospective study with only 18-month follow-up. To reduce bias, the study only included patients with type IIIB ACJ. This limited sample selection means that the results may not be generalizable to a broader patient population. Larger-scale studies involving more types and a greater number of patients are needed to further validate the findings. Moreover, histological studies are required to clarify the bone tunnel healing mechanism. Future research should incorporate multicenter randomized trials and finite element analysis to optimize implant design and validate the results in diverse populations. Also, long-term outcomes beyond 18 months, including implant durability and bone tunnel remodeling, require further follow-up.

## 5. Conclusion

LP-LTPS offers superior clinical outcomes with a short learning curve, fewer complications, better functional recovery, and early pain control compared to control group for Rockwood type IIIB ACJ dislocation, supporting its use as an effective alternative.

## Author contributions

**Conceptualization:** Liang Zhang, Lei Wang.

**Data curation:** Liang Zhang, Jin Zhang, Longhui Shao, Jing Li, Chunling Song, Juan Bai.

**Methodology:** Lei Wang.

**Software:** Jin Zhang, Longhui Shao.

**Supervision:** Lei Wang.

**Writing – original draft:** Liang Zhang.

**Writing – review & editing:** Lei Wang.
